# Time-Resolved Infrared Spectroscopic Evidence for Interfacial pH-Dependent Kinetics of Formate Evolution on Cu Electrodes

**DOI:** 10.1021/acscatal.4c03521

**Published:** 2024-09-03

**Authors:** Georgios Katsoukis, Hilbert Heida, Merlin Gutgesell, Guido Mul

**Affiliations:** Department of Chemical Engineering, MESA+ Institute for Nanotechnology, University of Twente Faculty of Science and Technology, Drienerlolaan 5, Enschede 7522 NB, The Netherlands

**Keywords:** electrochemical CO_2_ reduction, Cu electrodes, formate selectivity, FT-IR reflection−absorption spectroscopy, interfacial pH

## Abstract

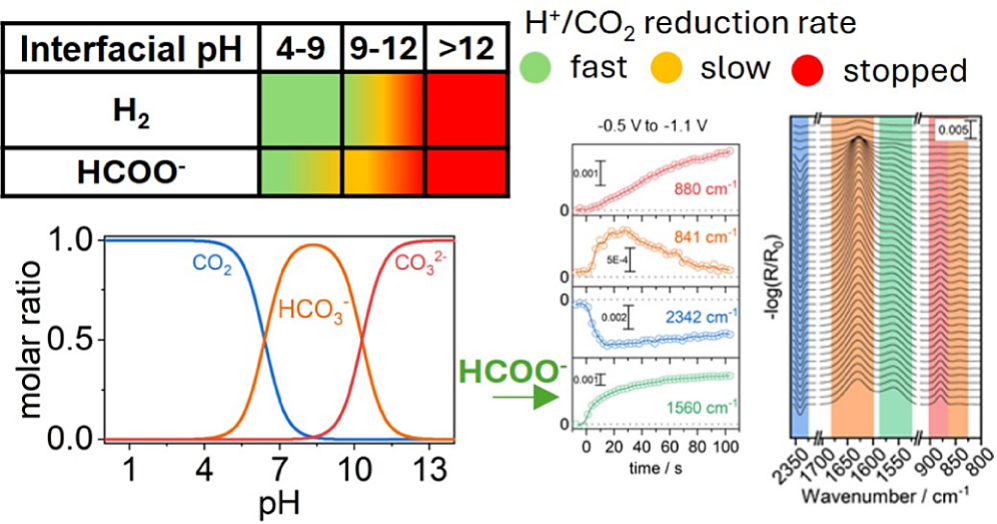

By deployment of rapid-scan (second time scale) electrochemical FT-IR reflection–absorption spectroscopy, we studied the reduction of CO_2_ in 0.1 M Na_2_SO_4_ in deuterated water at a pD of 3.7. We report on the impact of dynamic changes in the bicarbonate equilibrium concentration in the vicinity of a polycrystalline Cu electrode, induced by step changes in applied electrode potential. We correlate these changes in interfacial composition and concentrations of dissolved species to the formation rate of formate, and provide evidence for the following conclusions: (i) the kinetics for the conversion of dissolved CO_2_ to formate (formic acid) are fast, (ii) bicarbonate is also converted to formate, but with less favorable kinetics, and (iii) carbonate does not yield any formate. These results reveal that formate formation requires (mildly) acidic conditions at the interface for CO_2_ to undergo a proton-coupled conversion step, and we postulate that bicarbonate reduction to formate is driven by catalytic hydrogenation via in situ formed H_2_. Interestingly CO was not observed, suggesting that the kinetics of the CO_2_ to CO reaction are significantly less favorable than formate formation under the experimental conditions (pH and applied potential). We also analyzed the feasibility of pulsed electrolysis to enhance the (average) rate of formation of formate. While a short positive potential pulse enhances the CO_2_ concentration, this also leads to the formation of basic copper carbonates, resulting in electrode deactivation. These observations demonstrate the potential of rapid-scan EC-IRRAS to elucidate the mechanisms and kinetics of electrochemical reactions, offering valuable insights for optimizing catalyst and electrolyte performance and advancing CO_2_ reduction technologies.

## Introduction

Many research groups around the world focus on the development of CO_2_ storage and/or utilization solutions and technologies to mitigate climate change.^1^ Fortunately, the rapid growth in the generation of electricity by wind turbines or solar cells significantly reduces emissions of CO_2_. Moreover, CO_2_ can now be considered a feedstock for hydrocarbons, e.g., by utilization of electrochemistry (the CO_2_ reduction reaction, CO_2_RR).^2^

The CO_2_RR has been widely explored for a large variety of electrode materials in the past six decades, revealing trends and patterns, indicating that CO_2_ activation and C–C bond formation are not only intrinsically influenced by the composition, morphology, and interfacial structure of the electrode at both the nano- and mesoscales^3^ but also by a complex interplay between intrinsic electrode properties and extrinsic effects such as the dynamic composition of the interface and electrical double layer near the electrode surface.^4−6^ The electrical double layer is strongly influenced by electrolyte parameters such as pH, buffer strength, nature of cations/anions, and mass transport limitations.^7−9^

Independent of the electrode material, the thermodynamics and kinetics of both the CO_2_RR and the hydrogen evolution reaction (HER) change with (interfacial) pH. For the HER, the proton reduction reaction (2H^+^ + 2e^–^ ↔ H_2_) requires a lower overpotential than the water reduction reaction (2H_2_O + 2e^–^ ↔ H_2_ + 2OH^–^), which poses an additional barrier for an O–H bond of water to dissociate.^10^ Therefore, the HER can usually be suppressed when operating at high pH. However, the CO_2_RR usually requires a series of proton-coupled electron transfer steps, while the proton coadsorption energy on the electrode is predicted to determine the pathway toward specific products such as formic acid, CO, or more deeply reduced species.^11^ In addition, the CO_2_RR is strongly influenced by a sequence of (interfacial) pH-dependent homogeneous reactions occurring at the interface^12^:

1) Hydration of CO_2_ to bicarbonate (HCO_3_^–^ + H^+^) is slow and takes statistically around 27 s per CO_2_ molecule (*k*_hydration_ = 0.0371 s^–1^) at room temperature.
2) The reverse dehydration of HCO_3_^–^ to CO_2_ is also slow at pH 7 (ca. 16 s per CO_2_), but fast at pH 4 (ca. 16 ms per CO_2_) (*k*_dehydration_ = 2.67 × 10^7^ g·mol^–1^·s^–1^).
3) Hydroxylation of CO_2_ to HCO_3_^–^ (CO_2_ + OH^–^ ↔ HCO_3_^–^) is effectively an irreversible second-order reaction and is relatively slow at pH 7 (ca. 200 s per CO_2_) and fast at pH 11 (ca. 20 ms per CO_2_) (*k*_hydroxylation_ = 2.23 × 10^6^ g·mol^–1^·s^–1^; *k*_dehydroxylation_ = 9.71 × 10^–5^ s^–1^, > 1 day per CO_2_ molecule). The reaction of HCO_3_^–^ to CO_2_ will therefore proceed via dehydration.
4) Protolysis of HCO_3_^–^ to carbonate (HCO_3_^–^ ↔ CO_3_^2–^+ H^+^) and, inversely, protonation occurs in the nano- to microsecond regime (*k*_protolysis_ = 3.06 × 10^5^ s^–1^; *k*_protonation_ = 6 × 10^12^ g·mol^–1^·s^–1^).

These reactions primarily determine the time for the bicarbonate equilibrium to adjust to its local environment when it is out of equilibrium. Consequently, the time-scale and magnitude of the equilibrium shift will be determined not only by the rate of the HER (depending on applied potential) but also by the buffer capacity, ion effects, and mass transport – in particular advection – from the bulk electrolyte to the electrode interface. In addition, the CO_2_RR itself produces one hydroxide ion per electron transferred. The hydroxide ion produced at the interface will rapidly react with CO_2_ according to (3).^13^ A significant amount of interfacial CO_2_ will be depleted just by the buffering reactions, and increasing mass transport of CO_2_ to the electrode surface does not necessarily increase the CO_2_ reduction rate because of reaction (3).^14^

Understanding the complex interplay between spatiotemporally dependent acid–base bicarbonate equilibria and the intrinsic properties of electrode materials that undergo surface reconstruction is complicated. ATR-FTIR, Raman, and rotating ring disc electrode measurements have proven to be powerful techniques to obtain insights into the interfacial pH vs current densities (or applied potential) under a variety of CO_2_RR conditions at the electrode–electrolyte interface.^15−18^ Contrary to most studies advocating a high pH is necessary for effective conversion of CO_2_,^3^ in a systematic study that excludes the use of porous electrodes, a high faradaic efficiency of the CO_2_RR to CO on Au in a bicarbonate buffer was correlated to an interfacial pH needing to be relatively low, which is 6.8 in a CO_2_-saturated bicarbonate buffer.^19^ A low interfacial pH was able to be maintained using suitable cations (Cs^+^ > K^+^ > Na^+^ > Li^+^), high mass transport, and a high buffer capacity.^17,19−21^ These studies have focused mainly on steady state conditions, while transients in interfacial composition, in particular on short time scales (seconds), have received considerably less attention, since these are spectroscopically more difficult to resolve. We now elucidate how preconditioning of the Cu electrode, applied potential, and, most importantly, the dynamic changes of local concentrations impact the formation rate of products, in particular formate. We use a polycrystalline Cu electrode pretreated with multiple oxidative and reductive CV scans to obtain a steady electrochemically active Cu surface that does not undergo drastic changes upon sweeping or stepping of the potential. We apply rapid-scan electrochemical FT-IR reflection–absorption spectroscopy (EC-IRRAS) in a CO_2_-purged aqueous Na_2_SO_4_ electrolyte at pD 3.7 under stagnant conditions. This allows the observation of changes within the electrical double and diffusion layer. Dissolved CO_2_ is now resolved by IRRAS to be the primary reactant to form formate, while mass transport limitations induce a concentration change in dissolved CO_2_ to (bi)carbonate and eventually carbonate, which have a significantly lower contribution in the evolution rate of formate.

## Materials and Methods

### Materials

A Cu disk insert (5.0 mm OD, 4.0 mm thick) for ChangeDisk RDE setups was purchased from Pine Research, and 1 and 0.3 mm alumina polishing suspensions, deagglomerated, were purchased from Allied High-Tech Products. A standard Ag/AgCl reference electrode (3 M) was purchased from redox.me. Na_2_SO_4_ (>99%) and D_2_O (99.95%) were purchased from Aldrich. CO_2_ (>99.99%) was purchased from Linde.

### Electrochemical FT-IR Setup

A Bruker V80v was used equipped with an LN2 cooled medium-band (12000–600 cm^–1^) MCT detector, an MIR polarizer (KRS-5) inserted into an automatic polarizer rotational unit, which was set to p-polarization, an A530/V reflection unit for electrochemical cells, and a ZnSe transparent IR hemispherical crystal. Gold mirrors below the ZnSe crystal were set to an incident angle of 30 degrees, which results into another refraction at the ZnSe–water interface of around 70–80 degrees (depending on the wavenumber determined by the refractive index of water). This enables surface electric field enhancement through the “grazing angle”. A VersaSTAT 3 potentiostat was connected through BNC via a trigger connector box (Bruker, E525/Z) to allow triggering the potentiostat within the OPUS 3D software for synchronized IR vs *I*–*V* data accumulation. The resolution was set to 8 cm^–1^, and the aperture was set to 1.5 mm.

### Electrochemical FT-IRRAS Measurements (EC-IRRAS)

Prior to any measurement, the Cu disk and ZnSe IR crystal were polished and cleaned in an ultrasonic bath with distilled water. Before inserting all tools and chemicals into the IR spectrometer they were thoroughly dried to minimize any H_2_O contamination, since all experiments were performed in D_2_O. The electrochemical cell compartment was filled with 4 mL of electrolyte (see below for more details on the electrolyte), and the working electrode was inserted into a PTFE change disk to prevent any interaction of the side-walls of the electrode and placed upside-down (Otto configuration) onto the ZnSe crystal and aligned until a healthy signal could be obtained through external reflection. This was usually expressed by having a “thin enough” electrolyte layer of around 1.5–2 μm in between the electrode and the ZnSe crystal. The reference and counter electrodes were inserted into the electrolyte on opposite sides of the working electrode, respectively. Before purging the electrolyte with CO_2_, we performed 50 cyclic voltammograms at 0.2 V/s between +0.3 and −0.5 V vs RHE to oxidize, and desorb any contaminants from the Cu electrode, and condition the surface to obtain a stable electrochemical response. Then, the working electrode was lifted and reinserted into the electrolyte for mixing purposes and to prevent an IR response from contaminants formed near the Cu electrode (very intense signals were observed that could be assigned to C–H in D_2_O). Two types of experiments were performed: staircase linear sweep voltammetry (SCLSV) and chronoamperometry, using potential steps. The ohmic resistance was determined via electrochemical impedance spectroscopy 15 times, and the average of ca. 1000 Ohm was taken for correction in all cases. The resulting spectra were processed via OriginPro and baseline corrected using asymmetric least-squares smoothing with an asymmetric factor of 0.01 for positive peaks or 0.99 for negative peaks, a threshold of 0.0001, a smoothing factor of 9, and 10 iterations.

### Electrolyte Considerations

We used a 0.1 M Na_2_SO_4_ electrolyte that was set at pD 4 using concentrated H_2_SO_4_ (the amount of H^+^ in the solution is over 6 orders of magnitude smaller than D^+^). The electrolyte was purged for 30 min with CO_2_ until saturation, which resulted in a pD of around 3.7. We chose 0.1 M Na_2_SO_4_ at pD 3.7 saturated with CO_2_ over more conventional CO_2_-saturated 0.1 M KDCO_3_ (pD 6.8), so the evolution rate of the D-formate can be tracked over a wider pD range. The CO_2_ saturation concentration in a 0.1 M Na_2_SO_4_ solution was determined at pD 3.7 after continuous bubbling at 293 K and 1 atm (salinity = 14.204 g·kg^–1^), and amounts to 36.422 mM. This results into a bulk concentration of DCO_3_^–^ of 0.037 mM, and negligible amounts of CO_3_^2–^.^22^ The zero-point energy of D^+^ compared to H^+^ leads to elevated activation barriers and slower rates in the DER than in the HER. As a consequence, the quantitative results from our experiments might be different for experiments done under nondeuterated conditions. However, qualitatively, the observations will uphold as the concentration “trends” are similar in the HER and DER, both being promoted at lower pH(D).

### Definition of the Term “Interfacial pD”

From Figure S2, we estimate that the distance between the Cu electrode and the ZnSe crystal is approximately 1.5 to 2 μm. Around 10% of the vibrational intensities originate from within 6 nm of the electrode and over 40% from within 50 nm of the electrode as approximated by calculations performed in Note S2. From this, it can be concluded that the interfacial pD is determined at an average distance of around 100 nm.

## Results and Discussion

### Staircase Linear Sweep Voltammetry (SCLSV)

**Figure 1:**
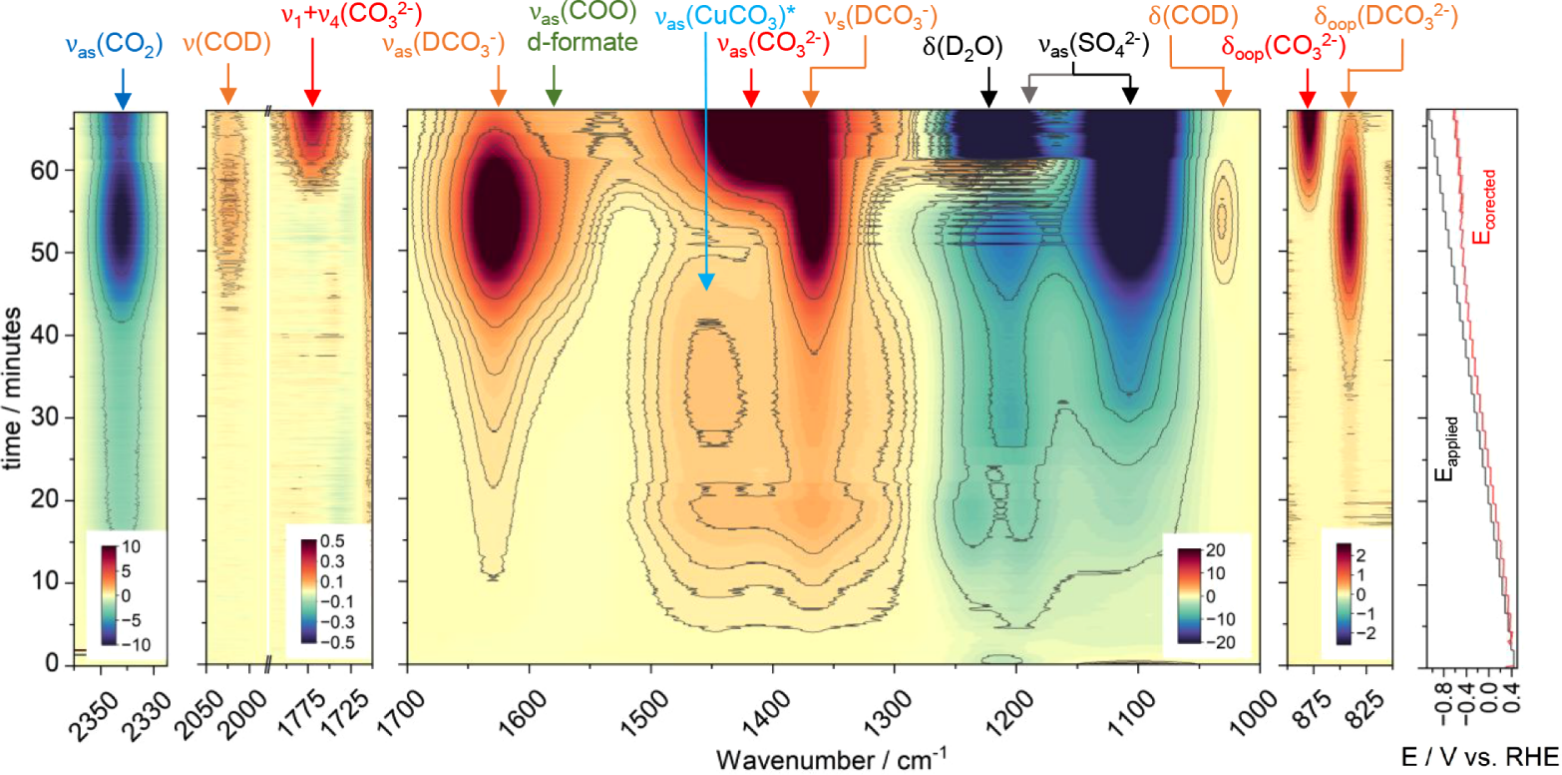
Contour plot of the ΔIR spectra–log(*R*_t_/*R*_0_) from +0.4 V to −0.6 V vs RHE (from bottom to top) in 50 mV steps using +0.5 V as a baseline. The step time was 128.1 s and 20 spectra (6.4 s/spectrum) were taken per step. The gray contours are for guidance. The scale bars are in ΔmOD and the intensity changes indicated by the change in color according to the legend – note the scale is different for various spectral ranges. The vibrational assignments (text) are color-coded per compound: dark blue is dissolved CO_2_, orange is dissolved D-bicarbonate, red is dissolved carbonate, green is D-formate, light blue is precipitated basic Cu*_x_*(CO_3_)_*y*_(OD)_*z*_, and black are deuterated water and sulfate. The time vs applied (black) and corrected (red) potentials including error bars are shown on the right.

To establish the methodology for determining the local pD before applying it to the rapid-scan measurements, we performed staircase linear sweep voltammetry for a CO_2_-saturated solution in 0.1 M Na_2_SO_4_ in D_2_O at an applied potential from +0.4 V to −1.1 V vs RHE using 50 mV steps, which led to an actual potential from +0.4 V to −0.6 V (Figure 1, right) after correcting for the pD change (−0.059 V pD) near the electrode interface.

Figure 1 shows a contour plot of the in situ IRRA spectra taken over time. A summary of the observed frequencies is shown in Table 1. The current–voltage profile can be found in (Figure S1. The CO_2_ asymmetric stretch is located at 2342 cm^–1^. The aqueous DCO_3_^–^ related bands are located at 841, 1031, 1365, 1628, and 2023 cm^–1^. The aqueous CO_3_^2–^ bands are at 880, (1050 cm^–1^ obscured) 1410, and 1769 cm^–1^. We also observed a broad band at around 1450 cm^–1^ evolving in the low overpotential regime from +0.0 V to −0.4 V vs RHE, which we assigned to the asymmetric carbonate stretch of basic Cu_*x*_(CO_3_)_*y*_(OD)_*z*_ – see below for further discussion. It is noteworthy that we did not observe any appreciable amount of CO_2_ reduction toward CO, despite of a noise level below 50 μ OD that allows us to time-trace the very weak C–OD stretch vibration of bicarbonate at 2023 cm^–1^ – discussion follows below. We can observe a weak shoulder appearing at 1560–1570 cm^–1^ at −0.6 V vs RHE, which we assign to dissolved deuterated formate (D-formate) – debate will follow further below.

**Table 1:** Assignment of Observed IR Bands

frequency (cm^–1^)	assignment
841	δ_oop_(CO_3_; DCO_3_^–^) aq.^25^
880	δ_oop_(CO_3_; CO_3_^2–^) aq.
979	ν_s_(SO_4_^2–^) aq.^26^
1103, 1194	ν_as_(SO_4_^2–^) aq.^26^
1217	δ(D_2_O) bend
1365	ν_s_(DCO_3_^–^) aq.^25^
1404	ν_s_(COO) D-formate monodentate tilted
1410	ν_as_(CO_3_^2–^) aq.^25^
1450	ν_as_(CO_3_^2–^) basic CuCO_3_ (Supporting Information)
1560	ν_as_(COO) D-formate monodentate tilted
1572	ν_as_(COO) D-formate aq.^27^
1628	ν_as_(DCO_3_^–^) aq.^25^
1770	ν_comb_(CO_3_^2–^) aq.^25^
2023	ν(OD; DCO_3_^–^) aq.^25^

The major spectral changes upon cathodic stepping can be traced back to

(1) removal of SO_4_^2–^ (i.e., asymmetric stretch doublet at 1106 and 1196 cm^–1^) from the beam path due to negative charging of the electrode,
(2) dynamic changes in the absorption region of D_2_O (i.e., bending mode at 1208 cm^–1^) due to molecular realignment along the electric field, which changes the interactions with p-polarized light, paired with a strong perturbation of the water network,^23^ convoluted within dispersion effects due to the large thickness of the “water film” that would require a Kramers–Kronig analysis to determine the actual absorption – we therefore omit the analysis of spectral features near (deuterated) water absorption,^24^ and
(3) shifting of the CO_2_/DCO_3_^–^/CO_3_^2–^ equilibrium from CO_2_ over DCO_3_^–^ (see strong positive intensities at 1628, 1365, and 841 maximizing at around 55 min) to CO_3_^2–^ (at 880 and 1410 cm^–1^, strong and still rising at 65 min) that originates from the reduction of protons (D^+^).

The results in changing bicarbonate and carbonate concentrations are largely in agreement with data reported based on the calculations^9^ and ATR-IR spectra^28^ of former studies by our group.

**Figure 2:**
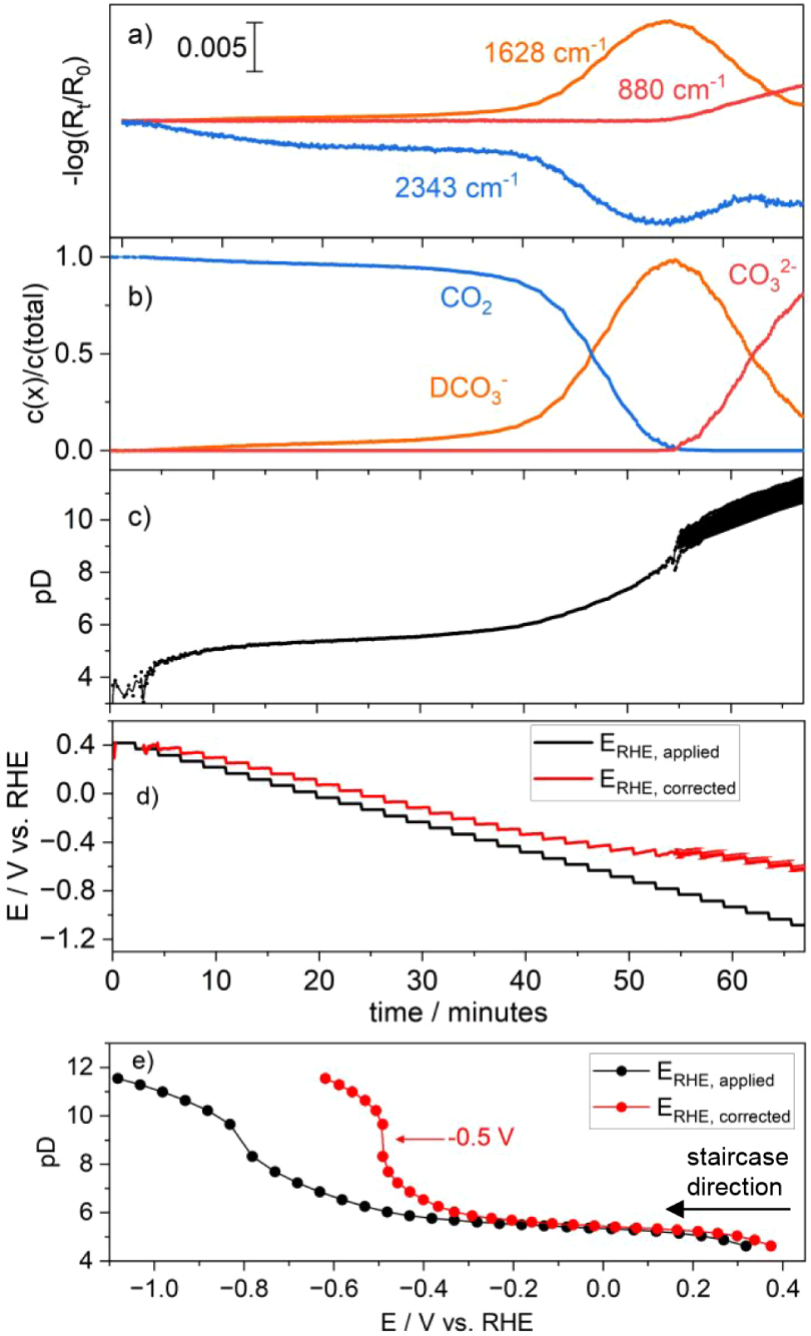
a) Intensity of dissolved ν_as_(CO_2_) at 2343 cm^–1^, ν_as_(DCO_3_^–^) at 1628 cm^–1^, and δ_oop_(CO_3_; CO_3_^2–^) at 880 cm^–1^ over time. Note that the Lambert–Beer law does not apply to the CO_2_ band intensity due to the OD stretch of D_2_O between 2250 and 2750 cm^–1^, blocking almost quantitatively the IR light. b) Calculated molar fraction of CO_2_ (blue), DCO_3_^–^ (orange), and CO_3_^2–^ (red) over time. c) Calculated near electrode pD over time and d) potential profile vs time (same as Figure 1, right). e) pD-corrected potential showing the typical Hendersson–Hasselbalch-alike sigmoidal pD change of a buffer solution at −0.5 V vs RHE.

The carbonate/bicarbonate system has been successfully used as a pH indicator for infrared spectroscopy.^29^ By tracing the intensity of the asymmetric stretch of DCO_3_^–^ at 1628 cm^–1^ and the out-of-plane bend of CO_3_^2–^ at 880 cm^–1^ (see Figure 2a), we can determine the molar fractions of CO_2_, DCO_3_^–^, and CO_3_^2–^ shown in Figure 2b. This, in turn, enables us to determine the local pD using the Henderson–Hasselbalch (HH) equation, see Figure 2c. See Note S1 for more details on the calculations. The error bars become large at pD values of above 10. Knowing the pD_t_ as a function of time allows us to correct the overpotential via *E*_RHE,corrected_ = *E*_RHE,applied_ – 0.059 pD_t_ – *iR* (*R* ≈ 1kΩ; due to the low currents, the ohmic resistance correction is almost negligible (<20 mV) even at a high overpotential). The HH equation can be applied because the equilibrium adjusts faster than the time-resolution of the experiment (see the Introduction section for the time constants). The local pD corresponds approximately to the average pD between the ZnSe crystal and the electrode, which has an estimated thickness of around 1.5 to 2.0 μm (see Figure S2), although we can expect some degree of signal enhancement of vibrational features closer to the surface, which shifts the midpoint closer to the electrode. Noteworthily, a change in local pD from around 4 to 5 occurs in the first 10 min in the potential regime from +0.4 V until +0.2 V vs RHE. We noticed that this initial pD change is sensitive to the electrode pretreatment conditions, attributed to the reduction of Cu_2_O to Cu (Cu_2_O + 2e^–^ + 2D^+^ → Cu + D_2_O). The pD remains stable between 5 and 6 until the onset potential for the DER at 40 min is at around −0.4 V vs RHE. Then, the depletion of CO_2_ and formation of bicarbonate take place buffering the pD until the capacity is used up at 55 min, leading to a swift increase in pD. The pD then starts to be buffered through the bicarbonate/carbonate equilibrium at around a p*K*_a2_ of 10.97.^29^Figure 2e shows the pD vs applied potential and corrected potential, which matches well the order of magnitude of the pH determined near the surface of an electrode using a variety of complementary techniques such as nonfaradaic probe reactions.^30^ Interestingly, only when the buffer capacity of the CO_2_/bicarbonate equilibrium becomes negligible does the corrected potential start to deviate strongly from the applied potential. This explains why the current is temporarily plateauing in *I* vs *E* curves (appears as a small peak/shoulder at around −0.4 to −0.7 V vs RHE in the CV) in dependence of the buffer strength, i.e. bicarbonate concentration.^31^

### Formate Evolution Vs Interfacial pD

**Figure 3:**
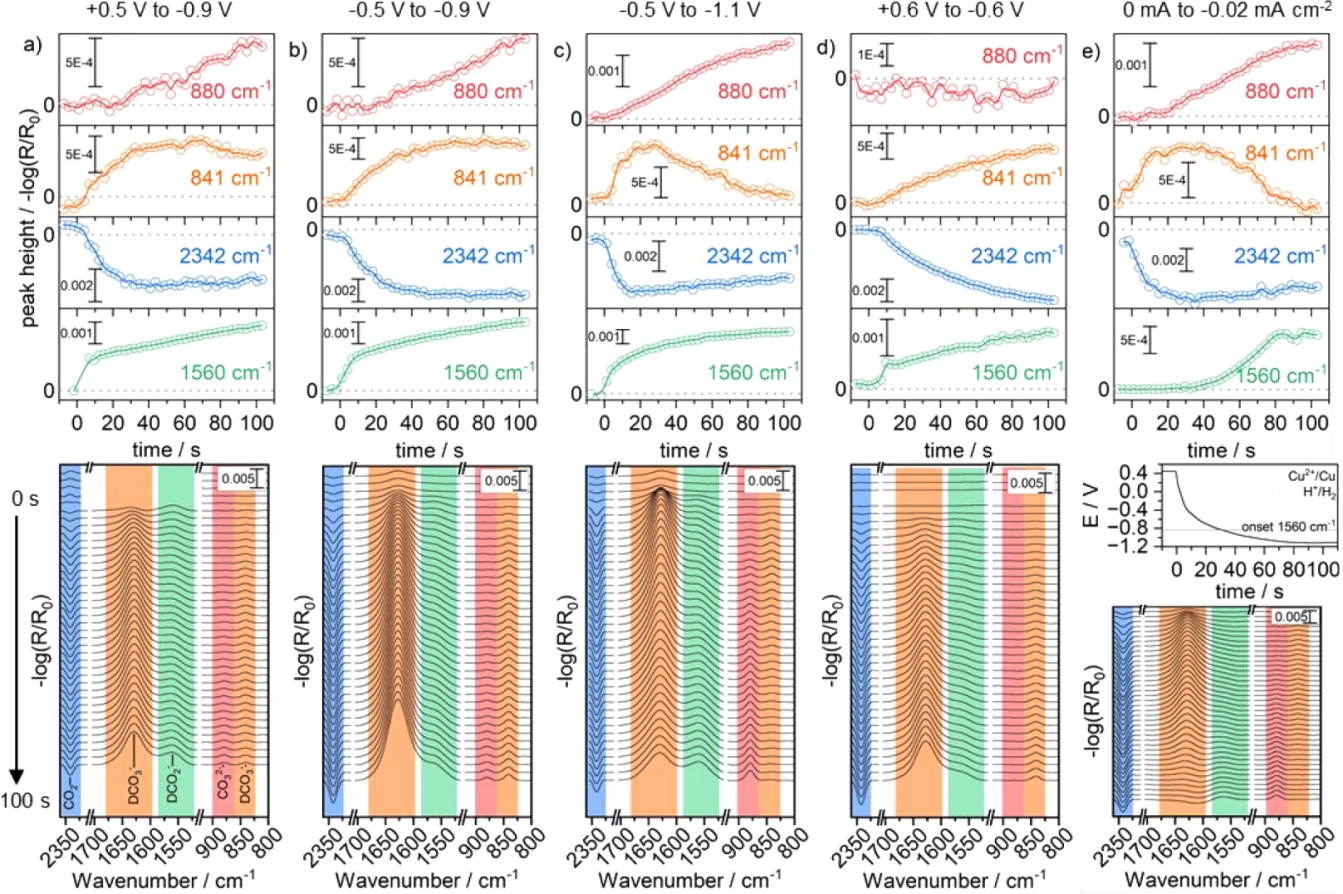
Time-resolved EC-IRRA spectra from 0 to 100 s showing the depletion of aqueous CO_2_ (blue), and the formation of aqueous bicarbonate (orange), aqueous carbonate (red), and D-formate (green): a) +0.5 V to −0.9 V, b) −0.5 V to −0.9 V, c) −0.5 V to −1.1 V, d) +0.6 V to −0.6 V, and e) chronoamperometry at −0.02 mA·cm^–2^ showing also the applied potential vs time profile. The lines indicate the standard electrode potentials of Cu^2+^/Cu, H^+^/H_2_, and the onset potential for the formation of D-formate (i.e., +0.8 V). Note that the “absolute” CO_2_ band intensity is reduced (here, ca. 0.005) due to the D_2_O OD stretch blocking most of the IR light. The formation rate of D-formate is fast at high concentrations of CO_2_, slow at high concentrations of D-bicarbonate, and absent at high concentrations of carbonate. The *I* vs *E* curves are provided in Figure S3.

Now that the spectral intensities have been correlated to potential-dependent local pD changes and concentrations of CO_2_, D-bicarbonate, and carbonate, respectively, we will discuss the time-dependent formation of formate in second-time resolution and correlate this to the main reacting species. We traced the evolution of D-formate over time (2.8 s per spectrum) using potential steps. Each experiment serves a purpose: in experiment (a), we step from +0.5 V to −0.9 V and probe the D-formate evolution starting from an oxidized Cu electrode; in (b), we step closely before the onset of the DER from −0.5 V to −0.9 V; in (c), we step from +0.5 V to −1.1 V toward a more negative potential; in (d), we step from +0.6 V to −0.6 V oxidative potential to the onset potential of D-formate; in (e), we apply a constant current profile. We applied different “pre”potentials to investigate the potential role of surface–adsorbate concentration on the initial kinetics once stepping toward a reductive potential. Figure 3 shows the rapid-scan infrared spectra for each experiment and the corresponding time traces at 840 cm^–1^ (bicarbonate), 880 cm^–1^ (carbonate), 1560 cm^–1^ (D-formate), and 2342 cm^–1^ (CO_2_).

Interestingly, in all chronoamperometry experiments (a) to (d), the formation of D-formate initially shows a rapid increase (see the green curve in Figure 3), before slowing down significantly at around 7 s and continuing at a slow, fixed rate. This transient in the kinetic regime coincides with the decreasing trend in the concentration of CO_2_, mirrored by the formation of D-bicarbonate. We can observe three noteworthy regimes, in which the formation kinetics of D-formate are different:

(1) When CO_2_ is abundantly available at the initial reductive potential applied, the formation of formate is maximized, as seen in all kinetic traces (Figure 3a–d) of the 1560 cm^–1^ band. This implies the presence of a CO_2_ pathway toward formate.

(2) When CO_2_ is depleted and bicarbonate is abundantly available, the formation of formate continues at a slower rate, implying that CO_2_ is not mandatory for the formation of formate but can proceed via a bicarbonate pathway.

(3) When bicarbonate is depleted, the formation of formate comes to a halt.

Regime (1) is located between 0 and 7 s in Figure 3a–d. Regime (3) is only visible in Figure 3c,e, where the 841 cm^–1^ band approaches zero at around 80 s, causing the growth of the 1560 cm^–1^ band to stop. Regime (2) is the intermediate phase, where the bicarbonate concentration plateaus.

The formation rate of D-formate is fastest before the onset of DCO_3_^–^, which points to the fact that CO_2_ is directly involved in the formate evolution mechanism and goes hand in hand with a previously proposed proton-coupled electron-transfer step to (adsorbed) CO_2_. It is debated if the formation of formate can also proceed via a direct conversion of bicarbonate.^32^ We observe that the formate evolution is not hindered upon depletion of CO_2_ at the interface and still evolves in the presence of bicarbonate but at a much slower rate. This suggests that both the CO_2_ and bicarbonate pathways are plausible, with the CO_2_ pathway being kinetically, significantly more favorable. At the onset of the formation of carbonate, D-formate evolution comes to a complete halt, indicating that carbonate is not a likely precursor for the formation of formate at the potentials applied here. This fact remains valid despite measuring an average pD at a distance of 100 nm from the electrode surface. Since deviations in the actual surface pD by 1–3 orders of magnitude might be possible, we cannot quantify the concentration overpotentials and determine accurate kinetic parameters for the reactions. However, this does not alter the fundamental observation that the formate formation rate is initially higher when CO_2_ is abundantly available and continues to evolve even after CO_2_ is depleted. It is worth noting that the initial surface–adsorbate concentration, which we attempted to tune through applying different “pre”potentials, does not have an effect on the initial formation kinetics of formate.

**Figure 4:**
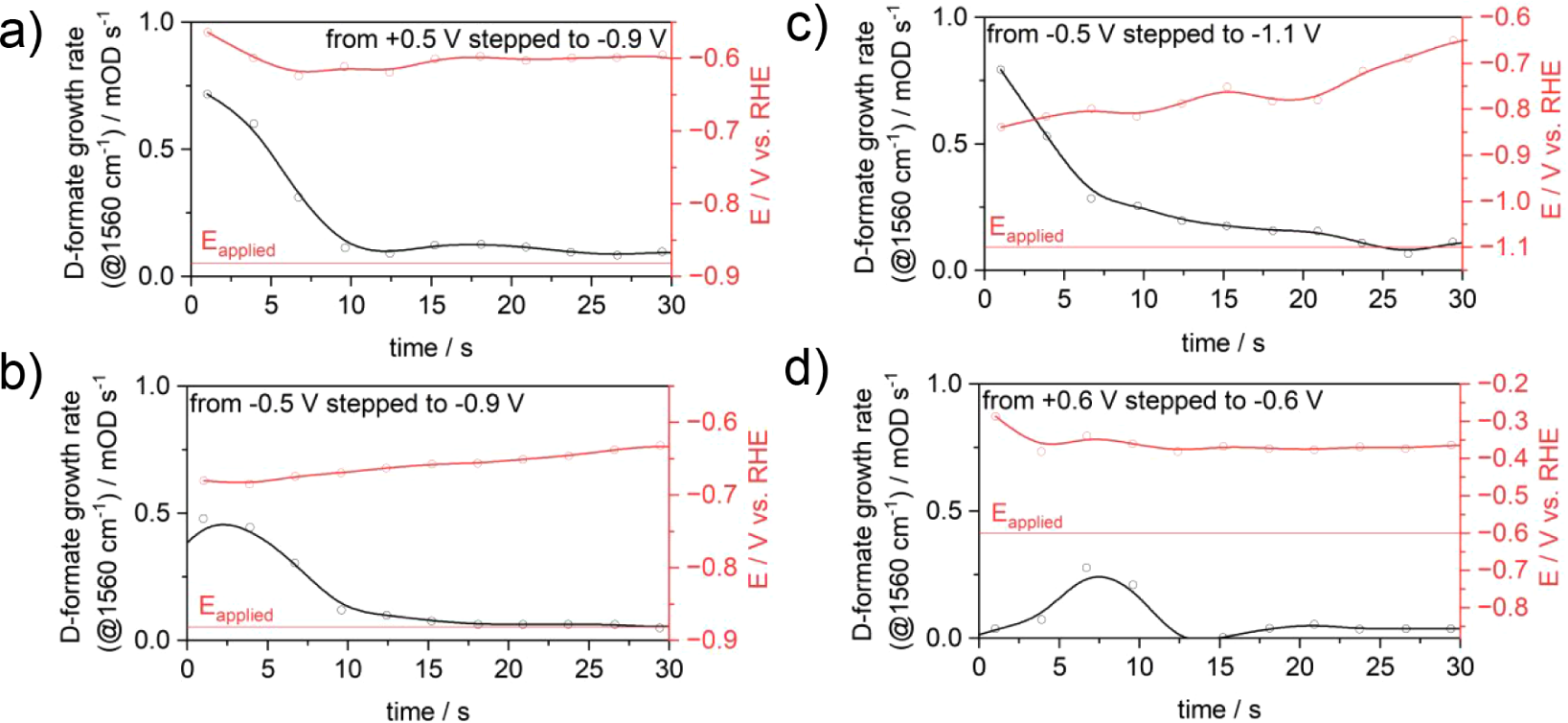
pD-corrected applied potential (red) vs the D-formate formation rate extracted from the growth of the band over time (1st derivative) at 1560 cm^–1^ (black). a–d) Inset shows the applied potential step. The red horizontal line shows the applied potential. The deviation of the pD and *iR*-corrected potential is in the range of 0.2 to 0.4 V.

To obtain a better understanding of the D-formate evolution kinetics, we need to understand quantitatively what the pD-corrected overpotential is near the interface to assess whether the faster formation kinetics are truly because of the CO_2_ or bicarbonate concentration at the interface, or can be traced back to a reduced thermodynamic driving force with increasing local pD. We plotted the formation rate of formate vs the pD and *iR*-corrected potential as shown in Figure 4. It can be seen that the deviation of the pD and *iR*-corrected potential is around 0.2 to 0.4 V less negative. We also observe that the overpotential vs RHE (red curve) is decreasing over time, reducing reaction rates. However, it is clearly noticeable that the D-formate formation rate (black curve) does not follow the same trend but shows, in all cases, a higher rate in the first 7 to 10 s. The initial fast rate, hence, is due to the abundant availability of near-surface CO_2_ in the initial stages of applying the reductive potential. The “prepotentials” applied, namely, +0.6 V (Figure 3d), +0.5 V (Figure 3a), and −0.5 V (Figure 3b,c), play an important role in ensuring that the initial concentration of CO_2_ is indeed maximized at the beginning. Figure 3e, for example, shows that formate evolution takes place when the HER is already ongoing, preventing the evolution of formate via the CO_2_ pathway, as it is already depleted by the shift in equilibrium. As a consequence, the equilibrium shift results into an increase in the Nernstian (concentration) overpotential for the reduction reaction. This concentration overpotential is indirectly connected to the interfacial pH, which governs the relative concentrations of CO_2_, bicarbonate, and carbonate. This supports the observations of the three kinetic regimes mentioned above, excluding an explanation via reduced overpotentials due to local pD shifts.^33^

These results imply that the kinetics of the electrochemical conversion of CO_2_ to formate are favorable under acidic conditions (pH ∼ 4), under which the competing HER is extensive for the applied Cu surface. It is obvious that all CO_2_ to formate conversion electrocatalysts reported in the literature are sluggish HER catalysts, because they consequently can maintain an acidic local pD.^11^ In addition, the faster kinetics, when preconditioning the electrode oxidatively (>+0.2 V vs RHE), reveals a possible explanation why pulsed and alternating current profiles can increase the production rates, since this way a more or less acidic interfacial composition (and associated relatively high CO_2_ concentration) can be maintained.^33^

**Figure 5:**
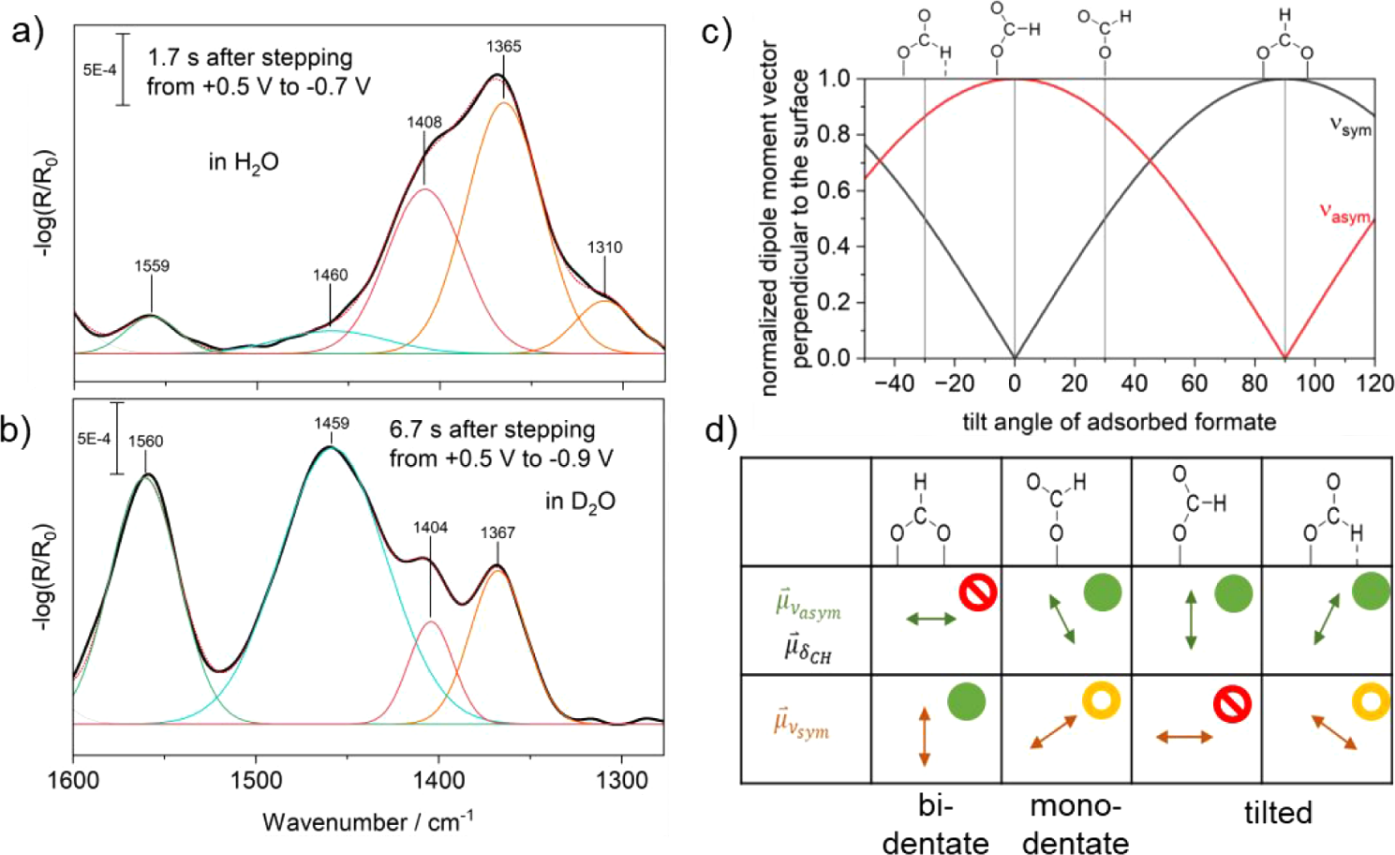
Deconvoluted EC-IRRA spectra after 1.7 s in H_2_O (a) and 6.7 s D_2_O (b) using +0.5 V vs RHE as a baseline. Orange are bicarbonate-related bands, red is carbonate, blue is CuCO_3_, and green is D-formate.^27^ c) Normalized dipole moment vector perpendicular to the surface vs tilt angle of formate, visualizing the allowed observable vibrational modes via IRRAS. d) Allowed vibrational modes (green), weekly allowed (yellow), and not allowed (red). Only the tilted adsorbed formate matches the observations. Note that for H_2_O, −0.7 V was applied to prevent the water bending mode from dominating the spectrum.

The underlying reason why CO_2_ predominantly converts to formate at acidic pH can be explained by taking a closer look at the band position of the asymmetric and symmetric stretch of the D-formate vibrations and the adsorption orientation. We assign the 1560 cm^–1^ band to the asymmetric stretch of the carboxy group of D-formate ν_as_(COO) (see Figure 5a,b).^27^ The asymmetric stretch for bidentate D-formate is invisible in p-polarized FT-IRRAS due to a dipole moment parallel to the surface (see Figure 5c). The asymmetric stretch for monodentate adsorbed D-formate in turn is usually located between 1600 and 1650 cm^–1^, which is too high to assign this vibration to the 1560 cm^–1^ band.^34^ Tilted adsorbed^35^ and aqueous^27^ D-formate match the position well and is usually observed at multilayer coverages and heterogeneous surfaces. For the tilted-adsorbed case, the ν_s_(COO) of D-formate is usually located at ca.^35^ 1360–1400 cm^–1^ but due to part of the dipole moment being in parallel to the electrode surface, it is very weak (Figure 5d). Therefore, we cannot deconvolute the signal from the bicarbonate symmetric stretch (1368 cm^–1^). We did not observe the postulated (O,O)-bidentate-formate within the time resolution of our experiment. We identified the carbonate asymmetric stretch (aq) at 1404 cm^–1^, which originates from the higher surface pD within the Helmholtz layer than the near-surface pD we determined in our experiment (100 nm). Note that the influence of the C–H(D) on both formate-related COO stretching vibrations are small upon isotope labeling, which cannot be captured by the resolution of our experiment (8 cm^–1^).^27^ The band at 1310 cm^–1^ in H_2_O can be assigned to the COH bend of bicarbonate.

It has been suggested via computational work by the group of Goddard^36^ – supported by theoretical works by the groups of Rossmeisl and Strasser^11^ – that the electrochemical reaction pathway for CO_2_ toward HCOO^–^ proceeds via physisorbed CO_2_ that reacts directly with a surface H* through a nucleophilic attack, which bypasses the initial activation of CO_2_ by the Cu surface. It was also speculated that this may be achievable by controlling the pH. The formation of a tilted-adsorbed formate suggests that CO_2_ hydrogenation indeed occurs through hydrogen adsorption on the electrode surface. The role of bicarbonate in the electrochemical CO_2_ reduction toward formate on Cu remains unclear. The direct catalytic hydrogenation of carbonate/bicarbonate in liquid phase via H_2_ is thermodynamically favored over the direct CO_2_ reduction,^37^ which is also favorable when using Pd catalysts.^38^ We speculate that the high H_2_ concentration in the vicinity of the electrode due to the HER may promote the hydrogenation of bicarbonate/carbonate. The slow kinetics of (bi)carbonate conversion on Cu may originate additionally from electrostatic repulsion from the electrode preventing an appreciable adsorption of bicarbonate and carbonate. More experimental work, similar to that applied for Pd electrocatalysts adding H_2_ or D_2_ to the CO_2_ feed gas, is needed to elucidate the bicarbonate reduction pathway on Cu.

### CO Evolution

It is worth dedicating a discussion to the absence of product CO under the experimental conditions applied. The formation of surface adsorbed CO in its different configurations can usually be observed at 2020–2070 cm^–1^.^28^ Our signal-to-noise is in the 0.01 mOD range, which means CO can be observed in the submonolayer regime.^39^ We can also exclude any preadsorption of CO on the Cu electrode at oxidative preconditioning potentials. At first glance, this is a surprising result considering the clear observation of CO in ATR-FTIR and Raman experiments.^28,40−43^ We observe two main differences in our experimental setup compared to what is commonly used in the literature: 1) in ATR-FTIR and Raman, a surface-roughened Cu electrode is used to be able to observe any bands through surface plasmon enhancement; 2) these methodologies are operated in the Kretschmann configuration which allows for significant mass transport toward the electrode by continuous bubbling of CO_2_ and forced advection. We exclude the surface roughening being responsible for the observation of CO compared to a polycrystalline Cu electrode, since it does not justify the absence of any CO in the 0.01 mOD regime. Instead, the swift pH change at the electrode interface in our configuration upon applying potentials more negative than −0.4 V vs RHE limits the availability of CO_2_ at the interface. From the experimental results, it is apparent that formate formation is very rapid, likely much more rapid than formation of CO, which does not instantly evolve, and likely requires constant supply of CO_2_ toward the interface. This coincides with the observation that CO poisoning of the HER is an important mechanism by which Cu maintains a high Faradaic efficiency for the CO_2_RR in aqueous electrolytes.^44^ As reported in the literature, at electrolyte flow rates of 2 mL/min CO appears after 7 s upon applying a potential of −0.7 V vs RHE, which is around the time CO_2_ is depleted in our configuration.^40^ Operating EC-IRRAS under a flow configuration is a challenging task that requires methodology development. We are currently testing the feasibility of microfluidic flow and radial flow cells.^45^

### Fast Formation of Basic Cu Carbonate Degrades the Cu Electrode in Pulsed Electrolysis

**Figure 6:**
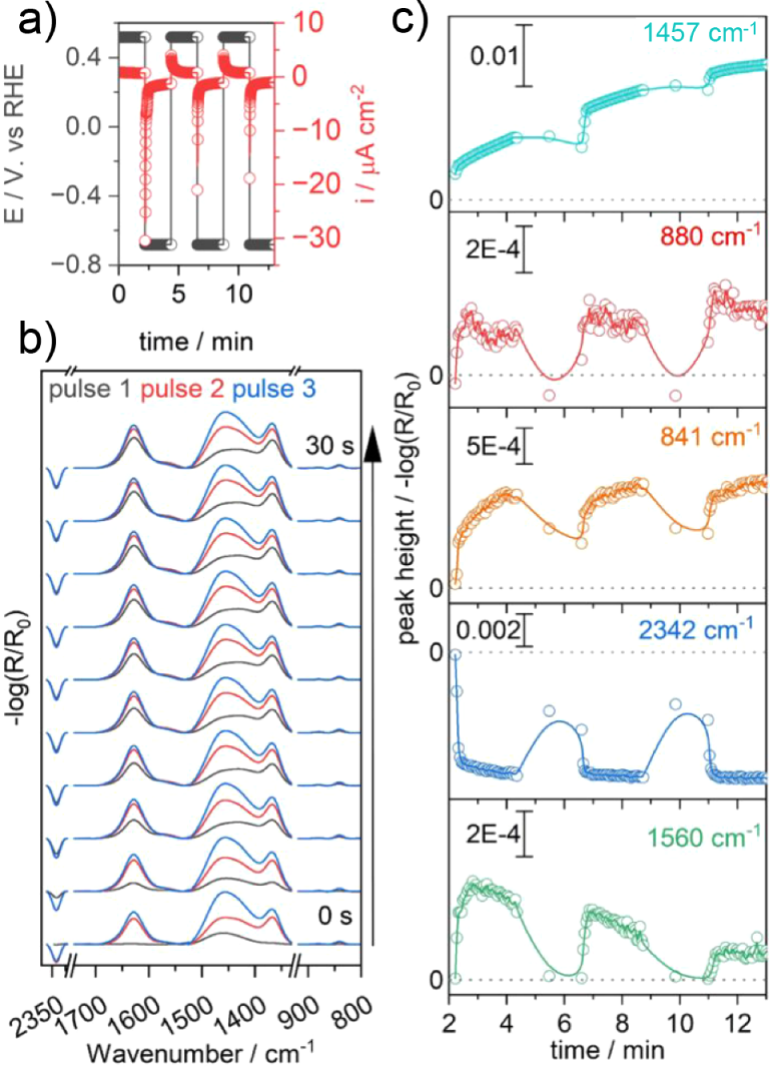
a) Applied square wave potential over time: 130 s −0.7 V vs RHE and 130 s + 0.5 V vs RHE (3 loops). b) EC-IRRAS spectra from 0 to 30 s taking point zero as the beginning of −0.7 V for each loop. c) Time traces for the evolution of the basic Cu carbonate (light blue, 1457 cm^–1^), carbonate (red, 880 cm^–1^), bicarbonate (orange, 841 cm^–1^), CO_2_ (dark blue, 2342 cm^–1^), and D-formate (green, 1560 cm^–1^).

We performed electrolysis using a square wave potential (3 loops) of 130 s at +0.5 V and −0.7 V vs RHE, with the idea to acidify the interface temporarily and eventually increase the yield of D-formate (see Figure 6). During the reductive applied potential, we observe the same features as in our previous chronoamperometric measurements. Upon reapplying +0.5 V, we can also observe that we are reacidifying our interface due to the recovery of the CO_2_ signal at 2342 cm^–1^. Unfortunately, we also reoxidize D-formate whenever we apply a positive potential of +0.5 V, which can be explained by the long residence time of D-formate in the vicinity of the Cu electrode in the stagnant electrolyte. In addition, the yield decreased with each loop. The only band that steadily increases is the band at 1457 cm^–1^, which we can assign to the asymmetric stretch (ν_as_(CO_3_, CuCO_3_)) of an amorphous malachite analogue that resembles the vibrational features of the mineral georgeite Cu_2_(CO_3_)(OH)_2_·6H_2_O.^46^ Through mixing of an aqueous CuSO_4_ solution with KHCO_3_ in water, we observe the formation of a Cu carbonate precipitate with a vibrational feature at 1465 cm^–1^ (see Figure S4). Basic Cu carbonate species at the interface have previously been observed as malachite- and azurite-type basic Cu carbonate. This also precipitates after long-term (multiple hours to days) or pulsed electrolysis experiments, due to Cu corrosion.^47^ In fact, we observed the evolution of this broad band at 1457 cm^–1^ within a few seconds. The p*K*_a_ of the reaction Cu^2+^ + HCO_3_^–^ → CuCO_3_ + H^+^ is 3.5, which means that Cu carbonates form also in the presence of bicarbonate at pH 3.5 or higher following the reaction Cu^2+^ + HCO_3_^–^ + 2H_2_O → Cu_2_(CO_3_)(OH)_2_(·*x*H_2_O) + 3H^+^.^48^ Alternating current electrolysis demonstrates, on the one hand, a fast degradation pathway of Cu due to the dissolution of Cu ions and reaction with bicarbonate toward basic Cu carbonate. On the other hand, we do not observe associated formation of formate. It is important to mention that formation of formate by conversion of basic Cu carbonate has been demonstrated recently by Lopez et al.,^49^ allbeit with a low Faradaic Efficiency of only 1%. Therefore, under our experimental conditions, the formation of formate will be insignificant.

## Conclusion

In conclusion, we successfully probed the electrochemical formation kinetics of D_2_ and D-formate with respect to the near Cu-electrode pD in a CO_2_ saturated aqueous (D_2_O) electrolyte via time-resolved electrochemical FT-IR reflection–absorption spectroscopy using p-polarized light. We used the bicarbonate and carbonate-related IR bands as an indicator to determine the local pD. Three interfacial regimes can be distinguished in which the formation kinetics of D-formate vary. The formation rate of D-formate is fastest at high abundance of CO_2_ at the interface, which points to the fact that CO_2_ is directly involved in the formate evolution mechanism. We observe that the formate evolution is not hindered upon depletion of CO_2_ at the interface and still evolves in the presence of bicarbonate, although at a much slower rate. This suggests that both the CO_2_ and bicarbonate pathway are plausible, with the CO_2_ pathway being kinetically more favorable whereas carbonate does not yield any D-formate at the applied potentials. The formation of a tilted-adsorbed formate suggests that the CO_2_ hydrogenation occurs through hydrogen adsorbed on the electrode surface. The conversion of CO_2_ to formate is therefore most favorable when the interfacial pH is acidic, likely around pH 4, to maximize CO_2_ concentration and minimize H^+^ concentration (see Figure S7 for more details on the hypothesis). The slow rate of bicarbonate conversion to formate suggests that hydrogenation of bicarbonate occurs catalytically (after formation of H_2_), but this requires further study.

The absence of any observation of CO can be traced back to the likely much slower kinetics in forming CO than that of formate under the applied reaction conditions. The formation of Cu carbonate has been demonstrated and is a possible degradation mechanism for Cu electrodes applied under experimental conditions for pulsed electrolysis.

## Supporting Information Available

Method description for local pH determination (Note S1); estimation of the distance from the electrode (Note S2); scheme of the spectroelectrochemical cell (Scheme S1); *iR* correction (Figure S1); quantification of D_2_O bending mode (Figure S2); *I*–*V* curves (Figure S3); FTIR spectrum of CuSO_4_ (Figure S4); quantification of the bicarbonate 1627 cm^–1^ peak (Figure S5); signal amplification vs distance from the electrode (Figure S6); hypothesis that a pH of around 4 may be ideal (Figure S7) (PDF)
