# Supplementary material for: Time-Resolved Infrared Spectroscopic Evidence for Interfacial pH-Dependent Kinetics of Formate Evolution on Cu Electrodes
Source: ACS Catal. 2024 Sep 3;14(18):13867–76. doi: 10.1021/acscatal.4c03521 (PMC11420947; doi:10.1021/acscatal.4c03521)
Supplement: Supplementary file 1 — cs4c03521_si_001.pdf [file cs4c03521_si_001.pdf]

SUPPLEMENTARY INFORMATION

Time-resolved infrared spectroscopic evidence for interfacial pH-dependent kinetics of formate evolution on Cu electrodes

Georgios Katsoukis,*[1] Hilbert Heida,[1] Merlin Gutgesell[1], Guido Mul[1]

Department of Chemical Engineering, MESA+ Institute for Nanotechnology, Faculty of Science and Technology, University of Twente, Drienerlolaan 5, 7522 NB Enschede, The Netherlands

* Corresponding author. E-mail: g.katsoukis@utwente.nl

Supplementary Note 1 (SI-Note 1):

To determine the local pD we proceeded as follows:

(1) The maximum intensity of the D-bicarbonate band at 841 cm^{-1} was taken as the (i.e. 0.00259±0.0003) anchor point that determines the maximum molar fraction of D-bicarbonate in the equilibrium which is 0.984. We then transformed the intensity at any given time to the molar fraction of D-bicarbonate through the simple mathematical calculation I_{841}/(0.00259*0.984). The molar fractions of CO2 and carbonate were then determined accordingly through 1=x(CO_2)+x(DCO_3^-)+x(CO_3^{2-}).

(2) Through the Hendersson-Hasselbalch equation we can calculate the pD=-log($[D^+]$) using the equilibrium constants K_{a1} and K_{a2}.

a. $$\frac{[DCO_3^-]}{C_T} = x(DCO_3^-) = \frac{[D^+]K_{a1}}{([D^+])^2 + [D^+]K_{a1} + K_{a1}K_{a2}}$$

b. $$[D^+] = \frac{\sqrt{K_{a1}}\sqrt{x(DCO_3^-)^2K_{a1} - 4x(DCO_3^-)^2K_{a2} - 2x(DCO_3^-)K_{a1}K_{a2} + K_{a1}} - x(DCO_3^-)K_{a1} +}{2x(DCO_3^-)K_{a1}}$$

c. The pD on the basis of the carbonate molar fraction was calculated as follows:

d. $$\frac{[CO_3^{2-}]}{C_T} = x(CO_3^{2-}) = \frac{K_{a1}K_{a2}}{([D^+])^2 + [D^+]K_{a1} + K_{a1}K_{a2}}$$

e. $$[D^+] = \frac{\sqrt{K_{a1}}\sqrt{x(CO_3^{2-})(K_{a1} - 4K_{a2}) + 4K_{a2}}}{2\sqrt{x(CO_3^{2-})}} - \frac{K_{a1}}{2}$$

The pD determined through carbonate has a larger error margin due to the logarithmic scale.

Supplementary Note 2 (SI-Note 2):

Determination of the distance from the electrode at which the pD is measured (see Scheme SI-1): In IR-reflection absorption spectroscopy, particularly with p-polarized light, the amplified electric field component normal to the surface decays exponentially away from the surface. The depth of this enhanced field into the bulk material typically extends only a few nanometers.[1] To estimate the distance at which the average pD is measured, we assume an exponential decay function for the electric field amplification of the form:

$$I_d(d) = I_\infty + I_0 \times e^{-\frac{d}{t}}$$

where $I_d(d)$ is the amplification factor of the intensity of a vibrational mode normal to the surface at distance d, I_∞ is the intensity amplification at infinite distance from the surface (set to 1 according to Lambert -Beer's law), $I_0$is the amplification factor right on the surface of the electrode (around 50), and t is the electric field decay constant.[1]

The absorbance of the asymmetric stretch of bicarbonate at 1628 cm^{-1} in Figure 1 at its maximum is 0.031 which corresponds to a concentration of 36.4 mM. Integrating that peak results in an area of 1.41 OD cm^{-1} – see SI-Figure 5. The extinction coefficient at 1627 cm^{-1} is around 614 M^{-1} cm^{-1} at a FWHM of 38 cm^{-1}.[2] Thus, the peak area extinction coefficient at 1627 cm^{-1} is 24,835 M^{-1} cm^{-2}. We can calculate the peak area for a transmission (non-surface enhanced) IR experiment using the Lambert-Beer law:

$A = \varepsilon cd = 24{,}835 M^{-1}cm^{-1} \times 0.0364\ \mathrm{M} \times 0.00085\ \mathrm{cm} = 0.77\ cm^{-1}$. 8.5 micrometer is the pathlength of the IR beam.

The area of the peak from Figure 1 is, thus, 1.41/0.77 = 1.8 times the intensity of a non-surface enhanced spectrum. Using now d = 1.5 micrometer for the thickness of the electrolyte layer in between the Cu electrode and the ZnSe crystal we obtain

$$\int_0^d [I_d(d)]\,dd = \int_0^d [I_\infty + I_0 \times e^{-\frac{d}{t}}]\,dd = 1.41$$

and

$$\int_0^d [I_d(d)]\,dd = \int_0^d [I_\infty\,]dd = 0.77$$

From which follows

$$\frac{\int_0^d \left[I_\infty + I_0 \times e^{-\frac{d}{t}}\right]dd}{\int_0^d [I_\infty\,]dd} = 1 + \frac{\int_0^d \left[I_0 \times e^{-\frac{d}{t}}\right]dd}{\int_0^d [I_\infty\,]dd} = 1 + \frac{\int_0^{1500} \left[50 \times e^{-\frac{d}{t}}\right]dd}{\int_0^{1500} [1\,]dd} = 1.8$$

The solution for the decay constant is 24 nm. SI-Figure 6 left shows the intensity amplification factor vs the distance from the electrode ratio of light absorbed with respect to the distance of the electrode using the aforementioned parameters. The average distance at which the pD is measured can be determined by integrating the function over the total distance and dividing it by the total distance. We estimate that the pD is measured at a distance of about 100 nm away from electrode which is closer than the midpoint distance between the electrode and the crystal (750 nm). The data shows that ca. 10 % of the IR signals are originating from within 6 nm of the electrode and 40 % from within 50 nm.

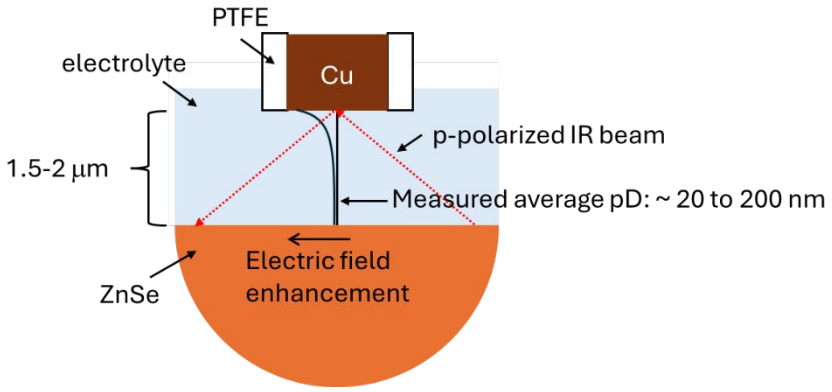

Scheme S1: Not-to-scale schematic of the area between the ZnSe-crystal and the Cu electrode depicting the position of the measured average pD in the results and discussion section. The electric field enhancement that leads to an amplification of IR vibrations up to 50 times at the electrode surface decays exponentially away from the Cu electrode.[3]

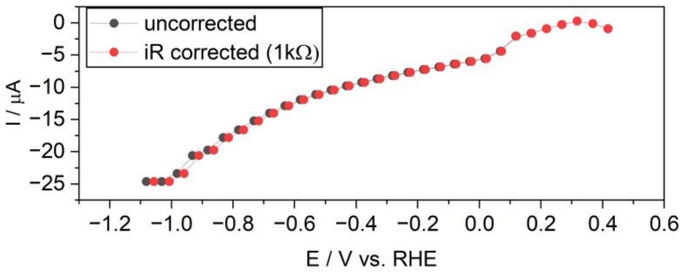

SI-Figure 1: I vs. E profile of the staircase linear sweep voltammogram discussed in Figure 1 of the main manuscript. Black is uncorrected, red is corrected by an ohmic resistance of 1kOhm. The influence of the ohmic resistance on the applied potential is below 25 mV even at high overpotential.

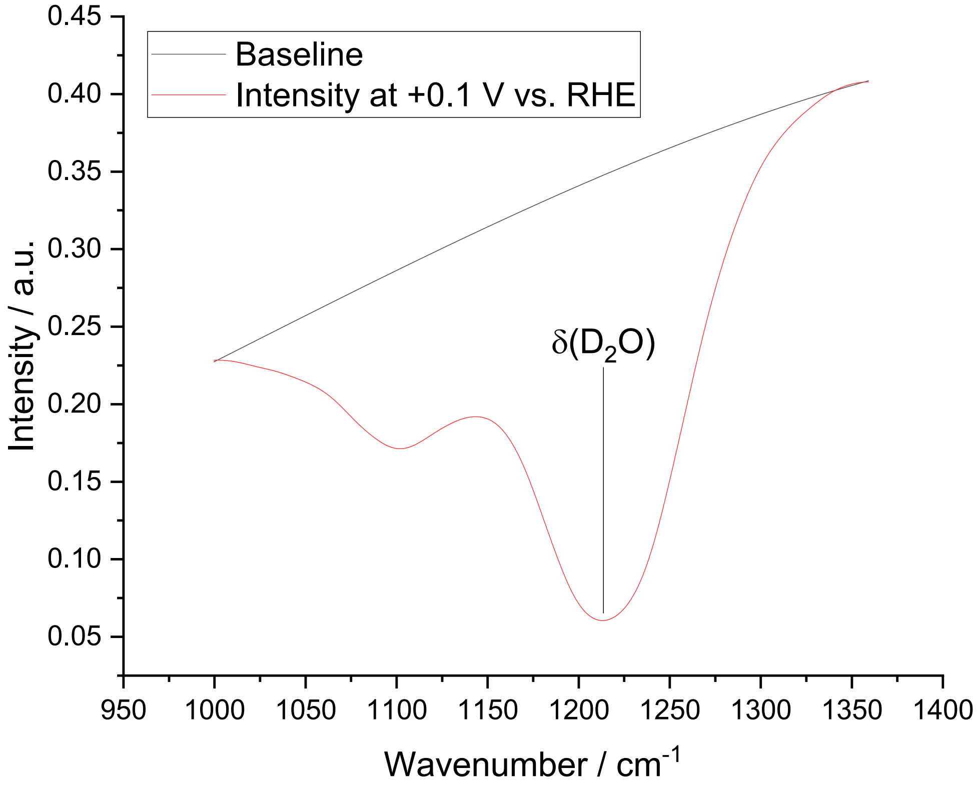

SI-Figure 2: Raw signal of the +0.1 V vs. RHE trace in the SCLSV measurement. Using an extinction coefficient of 16.1 cm^{-1} mol^{-1} for the D2O bending mode at 1220 cm^{-1}.[4] The pathlength is then calculated via: $\log\left(\frac{I_0}{I}\right) = \log\left(\frac{0.3496}{0.0614}\right) = 16.1\ cm^{-1}\ mol^{-1}\mathrm{L}\ * 55.14\ mol\ L^{-1} * length$. The pathlength is estimated to be 8.5 micrometer. Assuming a reflection angle of around 70±5 degrees the distance between the crystal and the electrode is estimated to be around 1.5 to 2 micrometer.

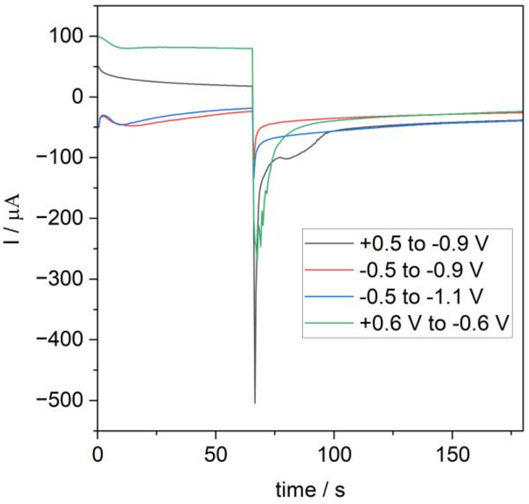

SI-Figure 3: Raw I vs. E curves for the chronoamperometric measurements in Figure 3.

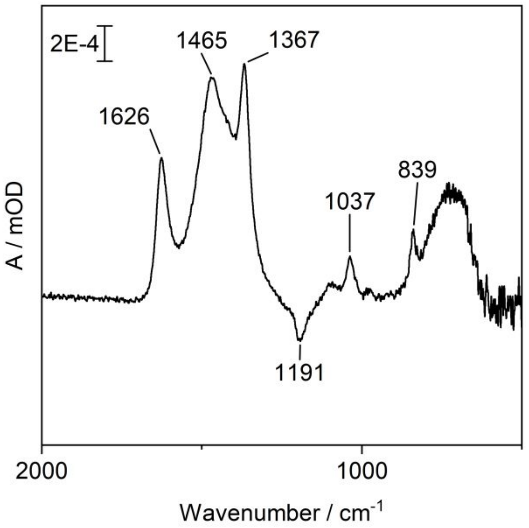

SI-Figure 4: ATR-FTIR spectrum of a $KHCO_3$ aqueous solution after adding a solution containing $CuSO_4$. The 1465 cm-1 band originates from the light blue coloured precipitate that instantly forms that can be assigned to basic Cu carbonate. All other bands can be assigned to bicarbonate.

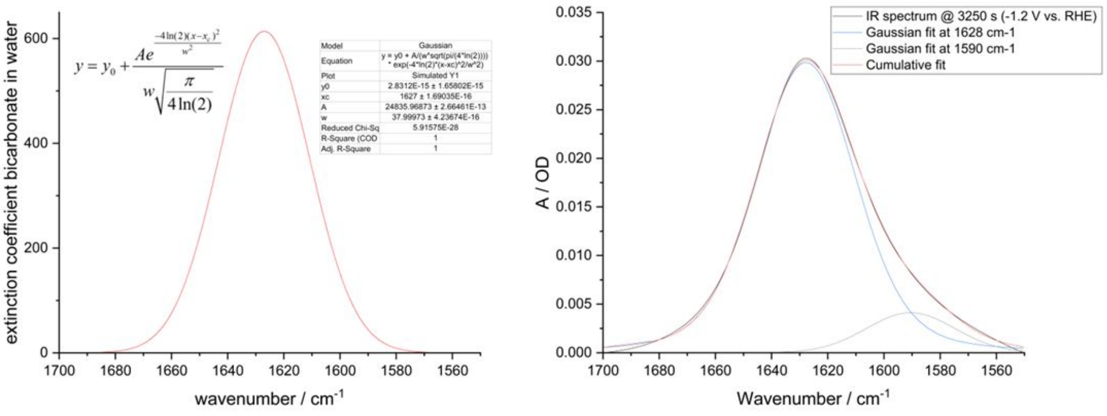

SI-Figure 5: Left: Simulated Gaussian curve of the bicarbonate peak at 1627 cm^{-1} as quantified by Barth et. al.[2] Right: Gaussian fit of the bicarbonate band at 1628 cm-1 for the IR-spectrum in Figure 1 taken at 3250 s. The peak area is 1.41.

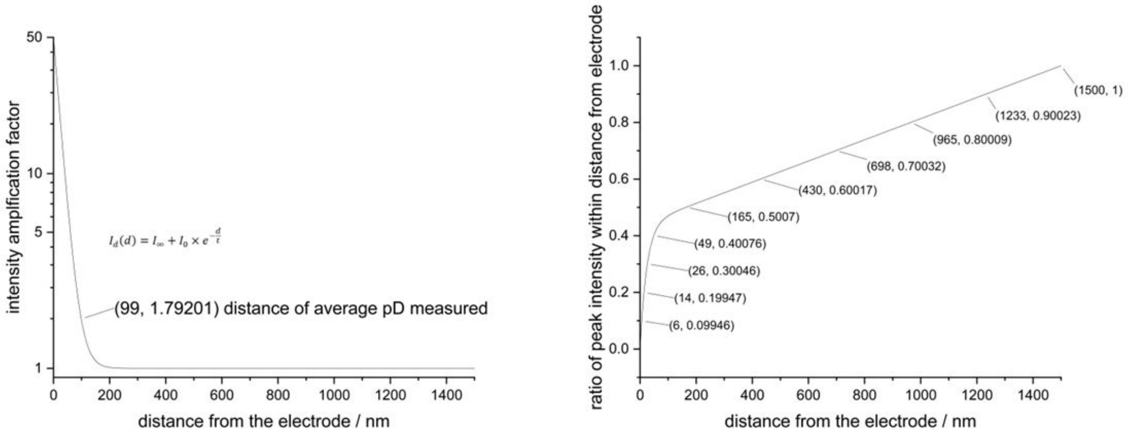

SI-Figure 6: Left: Amplification factor of IR vibrations vs. distance of Cu electrode using an exponential decay constant of 24 nm as approximated from supplementary note 2. Integration of the amplification factor across the whole distance between electrode and ZnSe crystal and subsequent division by 1500 nm yields the average distance at which the pD is measured in our experimental setup to a first approximation. It is around 100 nm. Right: ratio of peak intensity contribution within the distance of the electrode vs. distance from the electrode. The data points show that 10 percent of the signal are originating from within 6 nm of the electrode and 40 % from within 50 nm.

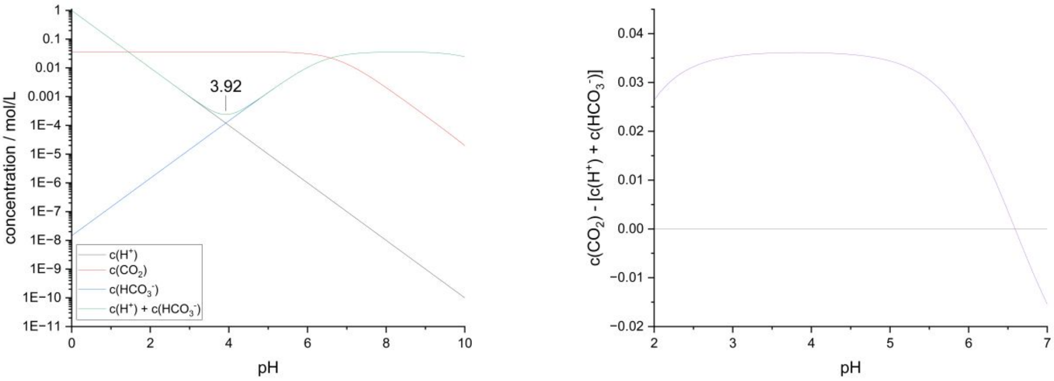

SI-Figure 7: Left: Bulk concentrations of protons, CO_2, bicarbonate, and the sum of protons and bicarbonate vs. pH were calculated using pK_{a1} and pK_{a2} values of 6.4 and 10.3, respectively, for the experimental conditions investigated. Considering that both protons and bicarbonate are effective proton donors for HER, the minimum concentration is at pH 3.92 to maximize the concentration overpotential for HER. On the right, it can be seen that while the ideal pH is at 3.92, the sensitivity is quite low, suggesting that a surface pH between 2.5 and 5 might be equally ideal for formate evolution. Notably, a 0.1 M bicarbonate solution saturated with CO_2 has a pH of 6.8, which is non-ideal in terms of concentration overpotential considerations. It is noteworthy to mention that the thermodynamic theory of a decoupled two proton-electron transfer predicts that the lowest thermodynamic overpotential occurs at the pKa of the reaction, which is 3.75 for formic acid.[5] At this point, it is unclear whether this is a coincidence coming from the fact that one hypothesis considers concentration overpotentials, and the other thermodynamic overpotentials.

(1) Chabal, Y. J. Surface Infrared Spectroscopy. *Surf Sci Rep* **1988**, *8* (5–7), 211–357. https://doi.org/10.1016/0167-5729(88)90011-8.

(2) Baldassarre, M.; Barth, A. The Carbonate/Bicarbonate System as a PH Indicator for Infrared Spectroscopy. *Analyst* **2014**, *139* (9), 2167–2176. https://doi.org/10.1039/C3AN02331A.

(3) Hoffmann, F. M. Infrared Reflection-Absorption Spectroscopy of Adsorbed Molecules. *Surf Sci Rep* **1983**, *3* (2–3), 107–192. https://doi.org/10.1016/0167-5729(83)90001-8.

(4) Bayly, J. G.; Kartha, V. B.; Stevens, W. H. The Absorption Spectra of Liquid Phase H2O, HDO and D2O from 0·7 Mm to 10 Mm. *Infrared Phys* **1963**, *3* (4), 211–222. https://doi.org/10.1016/0020-0891(63)90026-5.

(5) Koper, M. T. M. Theory of Multiple Proton–Electron Transfer Reactions and Its Implications for Electrocatalysis. *Chem Sci* **2013**, *4* (7), 2710–2723. https://doi.org/10.1039/C3SC50205H.
